# Hunting‐mediated predator facilitation and superadditive mortality in a European ungulate

**DOI:** 10.1002/ece3.3642

**Published:** 2017-11-23

**Authors:** Benedikt Gehr, Elizabeth J. Hofer, Mirjam Pewsner, Andreas Ryser, Eric Vimercati, Kristina Vogt, Lukas F. Keller

**Affiliations:** ^1^ Department of Evolutionary Biology and Environmental Studies University of Zurich Zurich Switzerland; ^2^ Zoological Museum University of Zurich Zurich Switzerland; ^3^ Carnivore Ecology and Wildlife Management KORA Muri Switzerland; ^4^ Federal Office for the Environment, Forest and Wildlife Biodiversity Section Ittigen Switzerland

**Keywords:** habitat selection, risk enhancement, step selection function, trophic interactions

## Abstract

Predator‐prey theory predicts that in the presence of multiple types of predators using a common prey, predator facilitation may result as a consequence of contrasting prey defense mechanisms, where reducing the risk from one predator increases the risk from the other. While predator facilitation is well established in natural predator‐prey systems, little attention has been paid to situations where human hunters compete with natural predators for the same prey. Here, we investigate hunting‐mediated predator facilitation in a hunter‐predator‐prey system. We found that hunter avoidance by roe deer (*Capreolus capreolus*) exposed them to increase predation risk by Eurasian lynx (*Lynx lynx*). Lynx responded by increasing their activity and predation on deer, providing evidence that superadditive hunting mortality may be occurring through predator facilitation. Our results reveal a new pathway through which human hunters, in their role as top predators, may affect species interactions at lower trophic levels and thus drive ecosystem processes.

## 1 INTRODUCTION

Predation risk is one of the key factors shaping animal space use patterns as prey species often have to trade off between finding enough food and being eaten (Lima & Dill, 1990; McNamara & Houston, 1987). Many prey species are eaten by more than one type of predator, resulting in a combination of threats that form a risk landscape through which animals have to move in order to acquire resources and reproduce (Sih, Englund, & Wooster, 1998).

Prey defense mechanisms depend on the predator‐specific hunting modes and the environmental context (Schmitz, 2008; Sih et al., 1998). For instance, avoiding an ambush predator may involve avoiding cover where ambush predators hunt most successfully, whereas avoiding an aerial predator implies avoiding open habitat. Predator‐specific prey defenses thus can lead to situations where avoiding one predator may increase the risk of being killed by another, a phenomenon known as predator facilitation (Charnov, Orians, & Hyatt, 1976). Although there are examples of predator facilitation from various systems in different taxonomic groups (e.g., Cresswell & Quinn, 2013; Fraser, Gilliam, Akkara, Albanese, & Snider, 2004; Kotler, Blaustein, & Brown, 1992), we do not understand how human hunters as top predators affect the susceptibility of their prey species to natural predators.

Not only prey alter their behavior in the presence of multiple predators. The activity and hunting strategy of predators themselves may depend on whether other predators feeding on the same prey co‐occur or not (Matsuda, Abrams, & Hori, 1993). For example, Embar, Raveh, Hoffmann, and Kotler (2014) showed experimentally that vipers (*Cerastes cerastes*) and owls (*Tyto alba*), both predators of gerbils (*Gerbillus pyramidum*), adjust their hunting activity depending on the presence of the other predator. By doing so, both predators may be able to improve their own hunting success. As a result, with predator facilitation, predation rates of two predators hunting the same prey combined may exceed the sum of the individual predation rates when only one predator was present (Sih et al., 1998).

Predator facilitation is thus a form of superadditive mortality, a term used in the hunting literature to refer to no‐hunting mortality indirectly caused by hunting (Kokko, 2001). Superadditive mortality has been shown to occur for a number of reasons such as suboptimal timing of the hunting season (Kokko, 2001), crippling losses, or when hunting disrupts the social structure of a population (Sandercock, Nilsen, Broseth, & Pedersen, 2011; Vucetich, Smith, & Stahler, 2005). However, superadditive mortality from hunting due to predator facilitation has rarely been investigated, despite the frequent presence of natural predators in harvested populations (e.g., Charnov et al., 1976; Melis, Nilsen, Panzacchi, Linnell, & Odden, 2013). Such additional hunting‐mediated mortality may have unexpected implications for the conservation of harvested species and may require adjustments to the hunting regime.

In this study, we quantify the degree to which the avoidance of simultaneously active hunters and natural predators results in predator facilitation and superadditive mortality in a lynx (*Lynx lynx*)‐roe deer (*Capreolus capreolus*) predator‐prey system. Roe deer are heavily hunted in many parts of Europe and they are generally the main prey of Eurasian lynx where these species co‐occur (Danilkin & Hewison, 1996). Lynx predation alone or in combination with hunting can have considerable effects on roe deer population dynamics depending on the environmental context (Belotti et al., 2015; Melis et al., 2013; Nilsen et al., 2009). Various studies have shown that roe deer adjust vigilance behavior and habitat selection to both human hunting (e.g., Benhaiem et al., 2008; Bonnot et al., 2013; Padie et al., 2015) and lynx presence (Eccard, Meißner, & Heurich, 2015; Wikenros, Kuijper, Behnke, & Schmidt, 2015). The hunting modes and environmental contexts of the two predators also differ: Hunting risk is highest in open areas during daylight hours, whereas lynx predation risk is highest in habitats with dense understory cover during twilight and night (Norum et al., 2015). Roe deer thus avoid hunters during the day and lynx during the night (Lone et al., 2017).

The goal of our study was to quantify the consequences of this trade‐off in predator avoidance for both roe deer and lynx and to shed light on the ecological role of human hunters as top predators in a multipredator‐prey system. We collected movement and activity data from 60 GPS‐collared roe deer (302'633 locations) and 13 GPS‐collared lynx (18'910 locations) and combined them with two independent data sets on seasonal mortality patterns of deer.

These independent sources of data allowed us to test the following three predictions: (1) the trade‐off between higher hunting‐related risk in the open and higher lynx‐related risk in the forest will increase overall exposure of roe deer to lynx predation risk during the hunting season. (2) Lynx will increase their activity during this period to benefit from increased prey susceptibility. (3) Increased prey susceptibility will facilitate lynx predation success and result in superadditive mortality for deer during the hunting season.

## 2 METHODS

### 2.1 Study area and hunting regime

**Figure 1:**
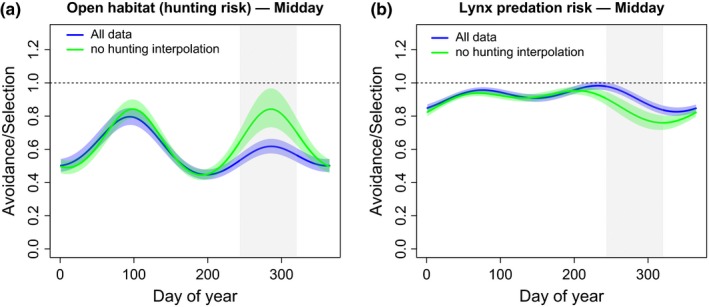
Contrasting risk avoidance of roe deer in response to hunting (a) and lynx predation risk (b). Blue curves show the avoidance/selection values (w(**x**) = exp(coef)) of the habitat selection model (SSF) using all data, whereas green curves indicate avoidance/selection for the no‐hunting interpolation. The color shaded areas denote the robust 95%‐pointwise confidence intervals for the all‐data model (blue) and the no‐hunting interpolation model (green), respectively. To visualize the effects, all covariates were set to their mean value except for open habitat (a) or predation risk (b). We fixed predation risk at the 75% quantile value (as an arbitrary proxy for high predation risk). Thus, the response shown denotes the avoidance of high predation risk (75% quantile) relative to the mean predation risk over the course of the year. Because we treated time of day (TOD) on a continuous scale, we fixed TOD at midday for visualizing avoidance/selection of hunting and lynx predation risk. The shaded area in gray depicts the 10‐week hunting period in the fall. The dotted line for w(**x**) = 1 represents no avoidance/selection

The study area in the Northwestern Swiss Alps (Appendix S1: FigureS1) covered roughly 1,500 km^2^ (center coordinates 46.559905 N, 7.513052 E) and ranged in altitude between 600 m and 3,500 m a.s.l. Most human settlements are situated at the valley bottoms. The hunting season on chamois *(Rupicapra rupicapra*) and red deer (*Cervus elaphus*) lasts all of September, whereas roe deer hunting occurs between October 1 and November 15. We considered the hunting period for all three ungulate species to be relevant, since the entire 10‐week period is characterized by an increased frequency of vehicular traffic and people patrolling in remote places, and previous studies have shown that hunting activities can affect nontarget species (e.g., increase in home range size or use of protected areas; Grignolio, Merli, Bongi, Ciuti, & Apollonio, 2011). In our study area, roe deer are shot almost exclusively in the open between sunrise and sunset (see below for details) using a sit‐and‐wait tactic, and hunting with dogs is very rare (B. Gehr personal observations). Lynx density in the region was estimated at 2.05 independent lynx/100 km^2^ in winter 2013/2014 (Zimmermann et al., 2014), and the main prey are roe deer and chamois (Gehr et al. 2017). Hence, hunting and lynx predation are the main causes of mortality for roe deer in the area (Gehr, 2016).

### 2.2 Available data and data preparation

#### 2.2.1 Movement data

Between November 2011 and April 2013, we captured deer (*n* = 60) using drive nets or box traps and equipped them with GPS collars (e‐obs GmbH, Gruenwald, Germany) recording locations every 30 minutes (*n* = 1,351,368 locations collected between December 2011 and March 2015). Because mean GPS error (27 m) was large with respect to the mean step length of 54 m (Visscher, 2006), we rarefied the data to 2‐hr fix intervals resulting in a data set with 302,633 deer locations (mean step length = 123 m). Simultaneously, 13 lynx were GPS‐collared in our study area. Locations were recorded on average every 3 hours yielding 18,910 GPS locations of lynx during the same period. Capture protocol and data collection for lynx are described elsewhere (Gehr, 2016).

#### 2.2.2 Mortality data

We analyzed two independent and temporally nonoverlapping data sets of deer mortality. The first data set stems from eight closely monitored GPS‐collared lynx in our study area in the period 2011–2015 (systematic search data), whose spatial clusters of GPS points indicating a kill were systematically searched for prey remains, resulting in 442 located lynx kills (of which 134 were roe deer). The second data set was provided by the cantonal hunting authorities and contained all reported cause‐specific mortalities of roe deer in the study area between 1990 and 2010 (public reporting data; *n* = 11,710). The vast majority of mortalities was due to human hunting (*n* = 8,099) and roadkills (*n* = 1,437). For the purpose of our study, we extracted the data on natural mortality (disease and starvation, *n* = 568) and lynx predation (*n* = 426; see below for a discussion on reporting bias). Note that this data set does not reflect the true frequency of mortalities, as human‐related causes (e.g., hunting and roadkills) are overrepresented due to much higher reporting probabilities.

### 2.3 Separating movement data of lynx and deer into active and passive states

#### 2.3.1 Roe deer

We expected the trade‐off between avoiding human hunters and lynx predation to be most pronounced when deer were active because most deer are shot while feeding in the open (see below). Reduced movement behavior, on the other hand, is considered an unspecific predator strategy likely effective for avoiding both human and natural predators (Lima & Dill, 1990). We thus restricted our analysis to phases of active roe deer behavior. We separated active and passive states based on tri‐axial accelerometer data (recorded every two minutes) and visually identifying an activity threshold using averaged accelerometer data. We then assigned each GPS location an activity state based on this information while verifying the analysis was not sensitive to the definition of our activity threshold (Appendix S1).

#### 2.3.2 Lynx

We predicted lynx to increase their activity during the hunting season (prediction 2). No accelerometer data were available for lynx to distinguish between activity states. We hence used a previously developed broken stick model (Gehr, 2016) to separate lynx steps into active and passive states based on movement speed (Appendix S1). Based on this model, we considered lynx to be active if they moved more than 0.77 m/min or 138 m in a 3‐hr step and passive otherwise (Figure S3). Visual inspection revealed that passive steps corresponded well with the cluster definition used to find kills and where evidence of feeding and resting was found during ground truthing.

### 2.4 Statistical analysis of prediction 1: Trade‐offs in avoiding humans and lynx

#### 2.4.1 Defining variables for modeling risk avoidance

We expected that deer trade‐off between high hunting‐related risk in the open and high lynx‐related risk in the forest (prediction 1). Over the course of the study, 13 of the monitored deer were killed by hunters, of which 11 were shot in the open and two were shot at the forest edge. Hence, we used open habitat (i.e., agricultural land, alpine meadows, settlement area, and rocky habitat) and forest as proxies for high and low hunting risk in modeling variation in risk avoidance and habitat selection of roe deer (dummy variable with 1 = open (high hunting risk)), and 0 = forest (low hunting risk)). As a proxy for predation risk, we used a previously developed resource selection function for active lynx (Gehr et al. 2017; Appendix S1), as lynx are more likely to be hunting while active. Therefore, we assume that predation risk is correlated with the probability of deer encountering an active lynx. In addition, we accounted for environmental variables previously shown to be important for roe deer in the area (Table S1). We included distance to the closest forest edge to account for the fact that deer may evaluate risk in the open depending on the distance to cover. We included house density as well as proximity to roads as proxies for human disturbance (Zimmermann & Breitenmoser, 2002). Furthermore, we included altitude and slope, as altitude correlates with climate variables, whereas steep slopes have been associated with low human activity (Basille et al., 2009; Zimmermann & Breitenmoser, 2002) but may pose increased risk from lynx (Lone et al., 2014). For both altitude and slope, a quadratic term was included to allow for nonlinear dependencies in habitat selection. Finally, southern exposed slopes (dummy variable with 1 = southern exposed slope and 0 = all other directions) may be preferred by ungulates during winter because this is where snow cover first disappears (Plank, 2013). Roe deer behaviors are not only governed by trade‐offs between different risks but also between risks and food acquisition (Brown & Kotler, 2004). Our approach implicitly takes resource selection, such as food availability, into account using the environmental variables (e.g., southern exposition, slope, or altitude) as proxies for the underlying biological drivers (e.g., food). However, an explicit test of trade‐offs between food acquisition and predation risk avoidance would require a different approach.

#### 2.4.2 Disentangling seasonal from hunting effects

All roe deer included in this study were exposed to hunting pressure, which precluded a comparison of hunted with nonhunted individuals in order to detect hunting effects. Comparing habitat selection during the hunting season to the period before and after is not practical, as seasonal trends in selection (e.g., a general decrease from summer to winter) and changes in behavior due to hunting (e.g., a short‐term increase) can be confounded. Instead, we built two separate habitat selection models using two nested data sets: the full data set which included all location data over the entire year (the all‐data model), and a reduced data set in which we excluded the 10‐week hunting period from the data and interpolated roe deer habitat use during the missing hunting period from the remaining data (no‐hunting model). This allowed us to compare habitat selection during the hunting period to that interpolated for the no‐hunting period while accounting for seasonal trends in habitat selection that otherwise masked effects of hunting. This method would fail to disentangle a seasonal from a hunting effect if the seasonal driver of habitat selection would perfectly coincide with the 10‐week hunting season. However, given the natural history of roe deer and the timing of the hunting season, we consider this scenario unlikely.

This approach was possible because we modeled seasonal variation in habitat selection and risk avoidance on a continuous time scale following the approach used in Forester, Im, and Rathouz (2009): We included interaction terms between open habitat, predation risk, altitude, and southern exposed slopes with four harmonics of day of year (DOY): s_1DOY_ = sin(2πt/365), s_2DOY_ = sin(4πt/365), c_1DOY_ = cos(2πt/365), and c_2DOY_ = cos(4πt/365). This is analogous to a reversed Fourier transformation, modeling a complex function of time using the first elements of a Fourier series where the period of the time harmonics determines the temporal scale under consideration (e.g., 365 for a year or 24 for a day).

Because human activity as well as lynx activity also differs between day and night, we further accounted for diurnal fluctuations of risk avoidance and habitat selection: We included additional interactions for open habitat and lynx predation risk as well as building density, distance to road, distance to forest edges, and slope with two harmonics of time of day (TOD: s_1TOD_ = sin(2πt/24), s_2TOD_ = sin(4πt/24)). This accounted for diurnal fluctuations averaged over the entire year and did not account for variations in day length (Forester et al., 2009).

All continuous covariates were standardized (mean of 0 and *SD* of 1). We used variance inflation factors (VIF) to test for multicollinearities between all model covariates. The highest VIF was 5.61 (predation risk), well below the threshold of 10 (Quinn & Keough, 2002). When excluding interaction terms with DOY and TOD, this VIF dropped to 2.65. There was no indication that the models were sensitive to the inclusion/exclusion of single predictor variables, hence multicollinearity did not seem to affect our models (Zuur, Ieno, Walker, Saveliev, & Smith, 2009). We further addressed this issue by producing out‐of‐sample predictions for our models (see section on cross‐validation below).

#### 2.4.3 Analysis of risk avoidance using step selection functions

To model trade‐offs in risk avoidance between humans and lynx, we built step selection functions using conditional logistic regression (SSF; Fortin et al., 2005). First roe deer paths were broken down into successive steps characterized by the step length (straight‐line segment between successive locations) and the turning angle (angle between previous and current step). Each step was then assigned the associated habitat variables and predation risk at the end of the step. We used 148,525 active steps for the all‐data model and 122,675 active steps for the reduced no‐hunting model. Each realized step of the final data sets was paired with 10 alternative random steps, which shared the same origin but had different end points. Random step lengths and turning angles were drawn in pairs from the empirical distributions in the data (Fortin et al., 2005). We included step length as a predictor in the regression analysis to account for characteristics of animal movement (Forester et al., 2009). To account for serial autocorrelation in the data, we calculated robust standard errors as described in Forester et al., 2009 (Appendix S1). Autocorrelation analyses indicated that autocorrelation could be neglected for lags beyond 9 steps (18 hr), considerably less than in comparable other studies (e.g., 3 days in wolves in Fortin et al., 2005 or 75 hr in elk in Forester et al., 2009).

To assess differences between the all‐data model and the reduced no‐hunting model, we calculated 95% effect displays for the predicted values under the different models using the robust covariance matrix as described in Appendix S1 (Forester et al., 2009; Fox, 2003). We emphasize that overlapping effect displays may still be statistically significant (Payton, Greenstone, & Schenker, 2003).

To compare the amount of observed variation in habitat selection explained by the different predictors, we assessed the relative importance of the different predictors in the SSF models using a resampling procedure as described in Ewald, Dupke, Heurich, Mueller, & Reineking, 2014 (Appendix S1). Finally, to assess the goodness of fit of our models, we performed k‐fold cross‐validation for a case–control design by leaving out animals as the test data set and using the remaining data as the training data set. As an additional test, we repeated the cross‐validation for a null model where we assumed a completely random pattern of habitat selection (Fortin et al., 2009; Appendix S1).

### 2.5 Statistical analyses of prediction 2: Increased lynx activity

To test prediction 2, we quantified the proportion of time lynx spent active while accounting for the same environmental covariates as in the lynx habitat model (Gehr, 2016; Table S2). We modeled the temporal fluctuations in the proportion of time lynx spent active using a logistic regression approach (P(active = 1)). To disentangle hunting from seasonal effects, we applied the same approach as for the SSF: One model with the full data set (all‐data model) and a reduced model where the hunting season was excluded (no‐hunting model). Furthermore, because we expected the strongest response from lynx while hunters are active, we restricted the analysis to the time between the beginning of astronomical twilight in the morning (sun angle < 18 degrees below the horizon) and the end of astronomical twilight in the evening (sun angle > 18 degrees below the horizon). In total, we used 11,469 lynx locations for the activity analyses. To capture temporal effects, we included interactions of covariates with harmonics of TOD and DOY in the same way as we did for the SSF models, except that we included main effects for the temporal predictors, which is not possible in SSF models. Autocorrelation analyses indicated that autocorrelation could be neglected for lags beyond 8 steps (24 hr). We then accounted for serial autocorrelation in the data using the NeweyWest function in the sandwich package in R (Newey & West, 1987). We performed model comparisons as described earlier by plotting 95% effect displays based on the robust covariance matrix.

### 2.6 Statistical analysis of prediction 3: Increased lynx predation success during the hunting season

We tested with the two independent data sets (see Mortality data) whether increased prey susceptibility during the hunting season facilitates lynx predation success, resulting in superadditive mortality of roe deer during the hunting season (prediction 3). We corrected estimates of numbers of roe deer killed by GPS‐collared lynx (systematic search data—see Mortality data) for fluctuations in sampling effort by dividing the detected number of roe deer kills by the number of monitored lynx each month (see Figure 3a for a depiction of the data distribution and the number of monitored lynx over time). This approach corrects for the temporal variation in lynx prey remain monitoring and hence for the variation in detection probability over time. We compared these direct estimates of roe deer kills by lynx (systematic search data) to estimates of reported lynx kills derived from the hunting authorities (public reporting data—see Mortality data and Figure 3b). We used a moving average window of size 31 days (i.e., 1 month) to remove zeros and get a smoothed function of the number of lynx kills over the course of a year (Shumway & Stoffer, 2011), before standardizing the moving averages to between 0 and 1 for statistical analyses. We modeled the seasonal variation in standardized lynx predation of roe deer in both data sets using a generalized additive model with identity link (GAM; Appendix S1). As before, we compared a full data model (all‐data model) with a reduced model (no‐hunting model) to disentangle hunting from seasonal effects. To quantify effect sizes, we compared the predicted occurrence of predation events during the hunting season of the two models and calculated the estimated percent increase in lynx predation due to hunting. In public reporting data, sampling effort and, hence, detection probability of lynx kills likely varied temporally because professional and recreational human activities in roe deer habitat that lead to the reporting of kills vary seasonally (e.g., hiking and hunting). In order to rule out that the observed pattern in predation rate (defined here as the number of kills per unit time) of public reporting data (Figure 3b) was an artifact of such variation, we repeated the analysis after correcting for seasonal variation in sampling effort or detection probability. To this end, we calculated the ratio between the reported number of lynx‐killed roe deer and the reported number of natural roe deer mortalities, assuming that sampling effort and detection probabilities for the two mortality causes are similar. This seems to be a reasonable assumption given that the public, which is reporting the mortalities, tends to visit all major habitat types where mortalities occur. Thus, when taking the ratio of the two causes of mortalities, variation in sampling effort and detection probability cancels out, making the ratio a proxy of the relative number of lynx‐killed roe deer that is independent of variation in detection probability and sampling effort. Note that, while sampling effort and detection probabilities of the two mortality causes are assumed similar, this approach does neither assume mortalities nor detection probabilities to be constant over time (see Appendix S1 for more details).

## 3 RESULTS

### 3.1 Prediction 1: Trade‐offs in avoiding humans and lynx

Avoidance of open habitat and lynx predation risk showed strong seasonal variation (Figure 1, Table S3). Comparisons between the all‐data model and the no‐hunting model revealed clear evidence that during the hunting season roe deer trade‐off risk avoidance from lynx and hunters. During the 10‐week hunting period roe deer avoided open habitat, where hunting risk is high, 24% more than they would if there was no hunting, at the expense of avoiding lynx predation risk less (12% at the 75% quantile for predation risk, see Figure 1). In contrast, there was no difference in selection/avoidance of altitude or southern exposed slopes between the all‐data and no‐hunting models (Table S3 and Figure S3).

Cross‐validation indicated that both models predicted roe deer habitat use well (mean Spearman rank correlations r_s_Hunt_ = 0.997, r_s_No‐hunt_ = 0.995) and were clearly different from the null model of random space use (Table 1). This high prediction accuracy from out‐of‐sample predictions indicates that overfitting was not a problem in our models despite the large number of predictors. Inspection of the relative importance of the different covariates in the two models showed that open habitat, distance to forest edge, and altitude were the most important predictors of deer habitat use (Table 1 and Table S4). The relative importance of lynx predation risk was very different between the all‐data model and no‐hunting model (9% in the all‐data model and 17% in the no‐hunting model), further demonstrating that during the hunting season roe deer trade off between risk from hunters and from lynx. For all other predictors, the relative importance was very similar between the two models (Table 1).

**Table 1:** Relative importance of the different habitat variables in the habitat selection model for roe deer (summed over the main effect and all interaction terms) together with the results for the cross‐validation analysis. Cross‐validation results represent the mean and range (in parentheses) of the Spearman rank correlations of 100 independent trials for used and random locations as described in Fortin et al. (2009)

	All‐data	No‐hunting interpolation
Habitat type	0.22	0.21
Predation risk	0.09	0.16
Edge distance	0.24	0.23
House density	0.05	0.04
Road distance	0.09	0.08
Slope	0.06	0.06
Altitude	0.21	0.18
Southern exposition	0.04	0.04
Sum	1	1
Cross‐validation_used_	0.998 (0.936, 1)	0.995 (0.918, 1)
Cross‐validation_random_	0.294 (0.000, 0.766)	0.277 (0.006, 0.851)

### 3.2 Prediction 2: Increased lynx activity

**Figure 2:**
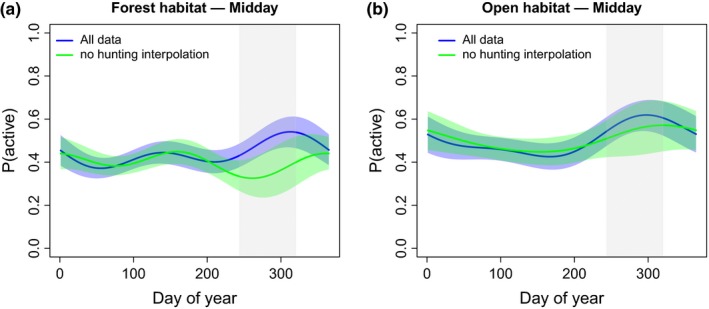
Contrasting activity patterns of lynx (a,b) over the course of the year. The results show the probability of a lynx being active inside the forest (a) and in the open (b) while setting all other covariates to their mean values. Blue curves show the activity for the all‐data model, green curves for the no‐hunting interpolation model. The color shaded areas denote the robust 95% ‐pointwise confidence intervals for all‐data (blue) and the no‐hunting interpolation models (green). The shaded area in gray depicts the 10‐week hunting period in the fall

During the hunting season, lynx increased their activity between dawn and dusk by 44% inside the forest, whereas activity remained unchanged in open habitat (Figure 2a,b; Table S5). In general, lynx were slightly more active when in the open than when in the forest.

### 3.3 Prediction 3: Increased lynx predation success

The two independent and temporally nonoverlapping mortality data sets showed broadly similar patterns of seasonal fluctuations in the number of roe deer killed by lynx (Figure 4a,b,d). The temporal patterns were also broadly similar with and without the correction for sampling effort and detection probability in the public reporting data (compare Figure 4b,d), although the amplitude of the peaks differed. The amplitude of the peaks also differed between data sets 1 and 2. However, both data sets and public reporting data with and without correction for sampling effort showed a peak in late winter, another peak during July/August (when juveniles start following their mother) and a third peak from September to November during the hunting season. In contrast, the seasonal patterns of natural mortalities in the public reporting data revealed no peak during the hunting season (Figure 4c), suggesting that natural mortalities did not occur more frequently during the hunting season. Furthermore, the lack of a peak in natural mortalities during the hunting season also indicates that the peak during the hunting season in the standardized ratio of number of roe deer killed by lynx divided by those that died of natural causes (our approach to correct for sampling effort and detection bias; Figure 4d) was caused by a change in the number of roe deer depredated by lynx and not by a change in the number of roe deer dying of natural causes. Overall, the estimated increase in predation rate during the hunting season was 55% in the systematic search data, 49% for the uncorrected predation rate in the public reporting data, and 50% for the corrected predation rate in the public reporting data. The independent mortality data sets and the different approaches to correct for sampling bias and detection probability thus yielded very similar results.

## 4 DISCUSSION

The aim of our study was to investigate the degree to which the trade‐off between avoiding hunters and natural predators results in predator facilitation and superadditive mortality in a lynx‐roe deer predator‐prey system. We found that roe deer avoided areas of high hunting risk during the hunting season at the expense of higher exposure to lynx predation risk (prediction 1). Lynx, in turn, increased their activity in the forest between dawn and dusk (prediction 2) and we found evidence of increased predation on deer during the hunting season (prediction 3). These results indicate that human hunting can induce predator facilitation through behavioral changes in both the prey and their natural predator, and we provide evidence that this predator facilitation resulted in superadditive mortality (i.e., additional indirect mortality caused by hunting). Given the frequent occurrence of natural predators in areas of harvested populations, we believe this topic merits further investigation in order to verify its generality and quantify its magnitude in other systems. In the following, we discuss in detail each of our three predictions.

### 4.1 Prediction 1: Trade‐offs in risk avoidance between humans and lynx

Our results show that a shift in habitat use as a hunting‐specific prey defense can lead to increased exposure to a natural predator in a hunter‐predator‐prey system (Figure 1). Roe deer clearly avoided open habitat during the day, and more so during the hunting season than would be expected from seasonal fluctuations in habitat preference. Shifts in habitat preference in response to hunting have been found in several species (e.g., Sunde, Olesen, Madsen, & Haugaard, 2009 for red deer, Said, Tolon, Brandt, & Baubet, 2012 for wild boar), including roe deer (Padie et al., 2015). In a similar context, Lone et al. (2017) found that roe deer in Norway exposed to hunters and lynx reduced their exposure to hunting risk during daylight hours while reducing exposure to lynx predation risk during the night. However, that study did not consider the response of the predator and the associated costs for the prey in terms of increased mortality. Here, we show that the observed hunting avoidance behavior of roe deer leads to increased exposure to lynx predation risk, which in turn resulted in increased predation pressure on deer (see below). These findings are in line with theoretical predictions that predators with different foraging modes or habitat selection (here: hunters in open habitat vs lynx in the forest) will provoke conflicting predator‐specific defenses, resulting in an overall risk increase for the prey (Sih et al., 1998). This increase in the overall risk landscape will not only result in altered space use but also affect existing trade‐offs between food acquisition and predation risk avoidance (Brown & Kotler, 2004).

### 4.2 Predictions 2 and 3: Increased lynx activity and predation success

**Figure 3:**
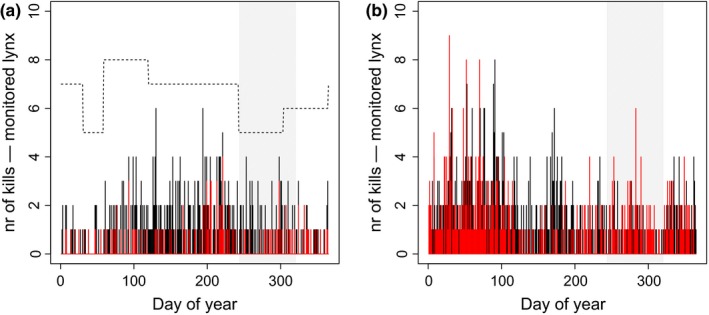
Sampling distribution of the number of roe deer killed by GPS‐collared lynx (a) and the number of lynx kills reported by the public in our study area over a 20‐year period (b). In (a), black bars represent the number of all prey items found during cluster controls on a given Julian day, whereas the red bars represent the number of roe deer kills found. The dashed line represents the number of lynx monitored every month. This value was used to account for sampling effort in the quantification of lynx predation on roe deer. In (b), black bars represent the number of reported roe deer natural mortalities on a given Julian day, whereas the red bars represent the number of reported lynx kills of roe deer. The shaded area in gray depicts the 10‐week hunting period in the fall

In addition to the specific behavioral response of the prey to changes in the risk landscape, we also found a behavioral response of the predator. There was evidence that lynx increased their activity between dawn and dusk during the hunting season in the forest but not in open habitat, perhaps to benefit from the increased prey susceptibility in the forest during this short time period (Figure 2). The observed increase in predation rate during the hunting season, apparent in two independent mortality data sets (Figure 3), supports this interpretation and links the behavioral shifts in roe deer to increased lynx predation as a result of predator facilitation.

**Figure 4:**
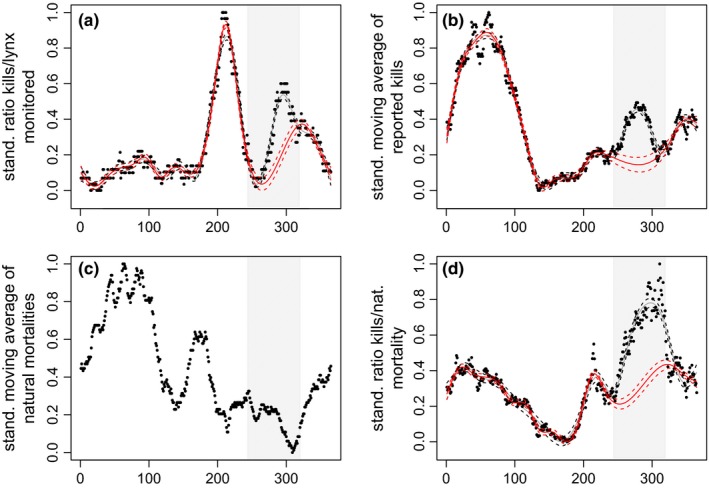
Seasonal fluctuations of the number of roe deer killed by lynx (a, b, d) or dying of natural causes (c). The solid black dots represent the standardized moving averages for the systematic search data corrected for sampling effort (a), the standardized moving averages of roe deer killed by lynx in the public reporting data (b), the standardized moving averages of natural mortalities in the public reporting data (c) and the standardized ratio between the number of roe deer killed by lynx and the number of natural mortalities in the public reporting data to correct for detection probability bias. The solid gray lines represent the predicted standardized number of roe deer killed by lynx from the generalized additive models (GAM), whereas the solid red lines represent the no‐hunting interpolation from the GAM's. Dotted lines represent the 95% confidence intervals around the GAM predictions. The shaded area in gray depicts the 10‐week hunting period in the fall

Comparisons of the two independent mortality data sets suggest that detection probabilities in these data sets did differ. The peak in late winter in both natural mortalities and lynx kills is much more pronounced in the uncorrected 20‐year data set of reported cause‐specific mortalities of roe deer in the study area (public reporting data; Figure 4b,c) than in the same data set corrected for seasonal variation in detection probability and sampling effort (Figure 4d) or the independent data set of roe deer killed by GPS‐collared lynx (systematic search data; Figure 4a). This indicates that this late winter peak likely resulted from an increased detection probability of carcasses during the cold period, either due to slower decay rates in the winter and/or increased recreational skiing activities in late winter. Similarly, the pronounced difference in the size of the mortality peak in July/August (compare Figure 4a,b), which coincides with the time when fawns start following their mother and thus fall more easily prey to lynx, can be explained with a much lower probability of small fawns being detected by a random observer (public reporting data) than by systematic searches of lynx GPS clusters (systematic search data). The latter have a very high detection probability even for small prey items (KORA, unpublished data).

The mortality peak during the hunting season in both data sets, however, is unlikely due to a detection bias. First, there is no peak in natural mortalities during the hunting season in public reporting data (Figure 4c), suggesting that the increased activity of hunters does not lead to a generally higher detection rate of carcasses during this period. Accordingly, the estimated increase in the number of roe deer kills by lynx during the hunting season was almost identical for public reporting data with and without correction for seasonal variation in detection probability and sampling effort (50% vs. 49%). Second, despite the completely different approaches to data collection in the two independent and temporally nonoverlapping mortality data sets (reporting of randomly encountered carcasses versus systematic searches for prey remains of GPS clusters of collared lynx), the estimated increase in predation during the hunting season is very consistent (between 49% and 55%). Taken together, this makes it likely that the peak during the hunting season reflects a true increase in lynx predation on roe deer during that time. Note, however, that we do not argue that the hunting season is the period with the highest lynx kill rate for deer. It clearly is not (Figure 4). Instead, our main conclusion is that there is evidence for a behavioral response of roe deer to hunting risk during the hunting season that leads to predator facilitation, which increases the risk of being killed by a lynx.

An alternative explanation for the increased lynx predation on roe during the hunting season could be that lynx abandon their kills prematurely in response to high hunter disturbance and therefore have to invest more time into searching and killing new prey, as has been found in cougars in California (Smith, Wang, & Wilmers, 2015). However, if this were the case, we would expect lynx to also increase their activity at night to hunt when human hunters are not active. We had no indication that this was the case (Figure S4). For these reasons, we believe that it is unlikely that the increased activity of lynx in the forest during the hunting season is a response to increased disturbance by hunters. Instead, the results likely reflect the behavioral plasticity of predators that enables them to capitalize on short‐term changes in prey vulnerability. Our findings thus suggest that predator facilitation may prompt predators to increase their activity in their preferred habitat in the presence of other predators that hunt the same prey (Embar et al., 2014). Furthermore, the results provide evidence that hunting‐mediated predator facilitation may cause superadditive mortality in harvested species. Such indirect effects have to be taken into account when assessing the overall impact of hunting on species interactions in ecological systems.

It is difficult to predict the population‐level effect of predator facilitation on both lynx and roe deer populations. Lynx have been found to kill roughly one ungulate per week (e.g., Molinari‐Jobin, Molinari, Breitenmoser‐Wursten, & Breitenmoser, 2002; Sunde, Kvam, Bolstad, & Bronndal, 2000). Thus, the roughly 50% increase in lynx deer kills we found during the hunting season implies that each lynx would kill five additional deer during the 10‐week hunting period. Given the lynx density in the area, this would add up to roughly 10 additional deer per 100 km^2^ (Zimmermann et al., 2014), a fairly small number even if deer densities were considerably lower than typical roe deer densities in Europe (Melis et al., 2009 reported a mean density of 1,046 deer per 100 km^2^ in areas with large predators—interquartile range 187–1,500 deer per 100 km^2^). However, short‐term and long‐term effects may be very different for both predator and prey in multipredator‐prey systems (Matsuda et al., 1993). Hunting may positively affect predator hunting success in the short run through risk enhancement as demonstrated here, however, if hunting depresses prey density over time, long‐term effects of hunters on both prey and predator may be negative (Sih et al., 1998). To better understand potential short‐term and long‐term consequences of hunting‐mediated predator facilitation, it will be necessary to quantify mortality rates of prey in areas with hunters and natural predators present alone or in combination with each other. Furthermore, a competing risk analysis with survival data of GPS‐collared deer could confirm superadditive hunting mortality due to predator facilitation from the prey perspective (Lunn & McNeil, 1995). Only an experimental approach would allow to test for the different trophic interactions between all players in such hunter‐predator‐prey systems.

## 5 CONCLUSIONS

Our habitat selection analyses for lynx and roe deer show that human hunting can alter the natural risk landscape of a prey, inducing behavioral changes in both predator and prey. Our subsequent analysis of the seasonal fluctuations of lynx predation on roe deer in our study site provides evidence that such hunting‐induced behavioral changes may result in superadditive prey mortality through predator facilitation. These findings have important implications for understanding the ecological role of humans as top predators driving lower trophic interactions in natural ecosystems. Moreover, such additional hunting‐mediated mortality may have to be taken into account by managers when setting hunting quotas for harvested species. Given the limited sample size of the predation data available in this study, we highlight the need for more detailed studies of harvested populations to determine the generality of our findings. Although we are aware of the difficulties of performing experimental studies in populations harvested by recreational hunters, we strongly recommend an experimental approach to shed light on hunting as an ecological force shaping prey population dynamics.

## CONFLICT OF INTEREST

None declared.

## AUTHOR CONTRIBUTIONS

BG and LFK designed the study. BG and MP collected the deer data. EJH, AR, EV, and KV collected the lynx data. BG performed the analyses and wrote the manuscript with contributions from LFK and KV.
