# Supplementary material for: Hunting‐mediated predator facilitation and superadditive mortality in a European ungulate
Source: Ecol Evol. 2017 Nov 23;8(1):109–19. doi: 10.1002/ece3.3642 (PMC5756843; doi:10.1002/ece3.3642)

**Electronic Supplementary Material Appendix S1**

**Hunting-mediated predator facilitation and superadditive mortality in a European ungulate**

Benedikt Gehr, Elizabeth J. Hofer, Mirjam Pewsner, Andreas Ryser, Eric Vimercati, Kristina Vogt and Lukas F. Keller

**Electronic Supplementary Material Appendix S1**

*Separate roe deer steps into active and passive states using tri-axial accelerometer data*

We separated location data from all deer into active and passive state based on tri-axial accelerometer data recorded for each deer. We first calculated 5 minutes acceleration averages (measured in millivolts) over all three axes and subsequently applied a running window of size two (averaging over two 5-minutes bursts) over all data for each deer. Then we plotted the moving acceleration averages against time and visually identified a threshold value which clearly separated active from passive phases for all animals (Figure S2). In our data set 10 millivolts clearly separated the data into these two states. Further increasing the threshold did not result in significant changes in activity states. Next we divided these moving averages into active and passive sequences based on the defined threshold value: an active burst started the first time the threshold was exceeded and ended the first time it fell below the threshold again. To avoid short periods of active behavior to be classified as a change in activity state (e.g. scratching while lying down and resting), we set the minimum length of an active phase to be 10 minutes. Again, shortening or prolonging this minimum length did not result in major changes in the duration of the activity bursts. Finally the time of the activity bursts was intersected with the time of the GPS fix to assign each location an activity state.

*Separate lynx data into active and passive states using a broken-stick model*

To separate lynx movements into active and passive states, we applied a broken-stick model based on speed (Sibly et al. 1990, Johnson et al. 2002). A two-process broken-stick model assumes that two Poisson processes generate movement rates. To separate the data into the two processes, we first categorized the movement rates into arbitrary regular spaces. Space length ranged between 0.1m/min and 2m/min in order to avoid empty categories that cause difficulties in the subsequent analysis of log-transformed data (see Sibly et al. 1990 for a detailed description). In a next step the log transformed frequencies of steps within each movement rate category were plotted against the movement rate categories (Sibly et. al 1990). A break point in the resulting curve shows the cutoff that separates the two processes that generate movement rates (Figure S3a). We determined the exact location of this break point using a simple maximization routine. Finally, we used equation 5 in Sibly et al. (1990) to calculate the optimal speed threshold for separating the two processes while minimizing the number of falsely assigned steps. GPS fix intervals ranged between one and six hours, with a majority of intervals at three hours (48%). Because we found that the movement rate was dependent on the fix interval (Figure S3b) we calculated two separate broken stick models, one for 1h fix intervals and a second one for longer fix intervals. The broken-stick model revealed an optimal threshold separating the fast and the slow process of 1.25m/min for the 1h GPS fix interval category, and 0.77m/min for the 3 and 6 hour categories (Figure 3a). In other words, if a lynx moved further than 75m in a one hour step or further than 138m and 277m in a 3 to 6 hour step, respectively, its behavior was categorized as active, and passive otherwise (see Gehr 2016 for a detailed description). Visual inspection revealed that passive steps corresponded well with the cluster definition used to find kills and where evidence of feeding and resting was found during ground truthing.

*Habitat selection model for lynx as a proxy for predation risk sensu Gehr 2016*

We used a resource selection function (RSF) for lynx while in active state as a proxy for predation risk for roe deer (Gehr 2016 and Table S2). We estimated lynx home ranges by drawing a buffer of twice the mean step length around the path of each individual lynx (all data pooled) and considered the resulting polygon as the home range of each lynx (Northrup et al. 2013, DeCesare et al. 2012). From this home range we drew 10 random locations per used lynx location. Finally we built the resource selection function using ordinary logistic regression. The RSF was of the form

w(x)= exp(β0 + β1x1+…+ βnxn)

where w(x) represents the RSF score, β0 is the intercept and the β’s are the regression coefficients for the corresponding covariates (x1,…, xn). Each random location was assigned a random time of day and day of year drawn from the observed lynx locations to account for the heterogeneous sampling scheme of GPS fixes.

*Calculating robust variance-covariance matrices for SSF model parameters sensu Forester et al. 2009*

To account for serial autocorrelation in the data we calculated robust standard errors as described in Forester et al. (2009, Appendix C). We first determined the lag at which serial autocorrelation of our SSF models declined below 0.05 and subsequently clustered the data according to the determined lag. We then created two data sets based on every other cluster and refit the original model to each of the two datasets using the cluster() function in the survival package in R. Finally we averaged the covariance matrices of the two models and used this robust covariance matrix to calculate confidence intervals for the regression coefficients calculated for the original model.

*Calculation of relative importance for the SSF models sensu Ewald et al. 2014*

We tested the importance of the different model predictors as described in Ewald et al. (2014) by first randomizing each predictor variable in turn and then building models including the permutated predictor. In the end the Pearson correlations between the predictions of the true model and the model with the permutated variable was calculated. Lower correlation values indicate greater importance. We inferred relative importance by subtracting each correlation coefficient from 1 and then scaled the results by dividing each by the sum of all those differences (Table S4). We repeated this procedure 100 times and reported the average relative importance over those trials. For assessing the overall importance for the main predictor variables we performed the rescaling after summing over all predictors including the particular variable of interest (spatial and temporal interactions included; Table 1 in the main text). However, we want to caution here, that this approach does not take collinearities between predictors into account and hence is not an exact measure for relative importance in the model.

*Crossvalidation approach sensu Fortin et al. 2009*

We performed k-fold cross-validation for a case-control design as described in Fortin et al. (2009) to assess the goodness of fit of our models. We repeatedly set aside 5 deer as the test data set and used the remaining data as the training data set. For the validation we ranked the w(**x**) score of the used location with respect to the random locations within each stratum (from 1-11) and subsequently recorded the frequency of occurrence of each rank within used locations. We then performed a Spearman rank correlation between the 11 ranks and the frequency of occurrence of each rank. We repeated this step 100 times, each time using a different training and test data set from all possible permutations (choosing 5 deer out of all deer with replacements) and reported the mean and range of r_s_. Following Fortin et al. (2009) we did the same for a null model where we assumed a completely random pattern of habitat selection. Instead of ranking the used locations against the random locations, we ranked a randomly selected random location within each stratum against the remaining random location in the stratum (1 out of 10, excluding the used location). The rest of the procedure was identical to the one described for the used locations.

*Modelling the seasonal variation in kill rates of lynx using a GAM*

We used the gam function in the gmcv-package in R (Wood 2011) to model the seasonal variation in kill rates of lynx . As for the SSF and the lynx activity model we compared a full data model (hunting model) with a reduced model (no-hunting model) where we omitted the data during the hunting season to interpolate the baseline kill rate in the absence of hunting. We did this for the two independent mortality datasets, the systematic search data and the public reporting data, which resulted in 4 different models. We chose penalized cubic regression splines for the smooth term of the GAM. In order to prevent model overfitting we chose a lambda value of 1.4 (Wood 2006, p. 224). We then tested model fit for 4 different values of dimension k (10, 12, 14, 16) in all 4 GAMs and chose the model with the smallest generalized cross validation score (GCV). The GAM model fit was generally good (Figure 3). For all 4 models the GCV scores for the different dimension parameters were very similar but the smallest GCV score was found for the dimension parameter k=16 in all cases (Table S6).

*Standardizing the lynx predation rate of the public reporting data set*

In order to rule out that the observed pattern in the predation rate of the public reporting data was an artefact of temporal variation in sampling effort or detection probability, we repeated the analysis of lynx predation rate after correcting for seasonal variation in sampling effort or detection probability. To this end we calculated the ratio between the reported number of lynx-killed roe deer and the reported number of natural roe deer mortalities. Using the ratio of number of lynx kills divided by the natural mortalities does neither assume that natural mortalities are constant over time nor does it assume that the detection probabilities are constant over time. It does assume, however, that the detection probabilities of lynx kills and natural mortalities are the same. In brief, if p_l, C_l, N_l, p_nm, C_nm, and N_nm are the detection probabilites and observed and true numbers of lynx kills and natural mortalities, repsectively, then C_l/C_nm = (p_l*N_l)/(p_nm*N_nm). The ratio of observed lynx kills and natural mortalities (C_l/C_nm) is an unbiased estimate of N_l/N_nm if p_l = p_nm. Note that the detection probabilities and different mortalities are free to vary over time. One would simply need to add an additional subscript for time to the above equation. The only assumption is that p_l = p_nm at all times. Because this seems a reasonable assumption, taking the ratio between these two forms of mortality allowed us to correct for differences in detection probability before, during and after the hunting season. Note that any fluctuation in the corrected kill rate of the public reporting data could be caused by changes in lynx predation, changes in natural mortality, or both. In order to show that the observed pattern is truly due to increased lynx predation, we show in Figure 4 of the main text in addition to the corrected predation rates (Figure 4d) both the uncorrected predation rates (Figure 4b) and the natural mortalities (Figure 4c), which we used to correct for detection bias. If the peak in predation rate during the hunting season would be solely caused by an increase in detection probability of kills, this would also be apparent in the natural mortalities. Since this is not the case, we believe this is good evidence that the observed pattern is truly caused by increased lynx predation.

**References**

DeCesare, N. J., M. Hebblewhite, F. Schmiegelow, D. Hervieux, G. J. McDermid, L. Neufeld, M. Bradley, J. Whittington, K. G. Smith, L. E. Morgantini, M. Wheatley, and M. Musiani. 2012. Transcending scale dependence in identifying habitat with resource selection functions. Ecological Applications 22.

Forester, J.D., Im, H.K. & Rathouz, P.J. (2009). Accounting for animal movement in estimation of resource selection functions: sampling and data analysis. *Ecology*, 90, 3554-3565.

Fortin, D., Fortin, M.-E., Beyer, H.L., Duchesne, T., Courant, S. & Dancose, K. (2009). Group-size-mediated habitat selection and group fusion-fission dynamics of bison under predation risk. *Ecology*, 90, 2480-2490.

Gillies, C. S., M. Hebblewhite, S. E. Nielsen, M. A. Krawchuk, C. L. Aldridge, J. L. Frair, D. J. Saher, C. E. Stevens, and C. L. Jerde. 2006. Application of random effects to the study of resource selection by animals. Journal of Animal Ecology 75:887-898.

Ewald, M., Dupke, C., Heurich, M., Mueller, J. & Reineking, B. (2014). LiDAR Remote Sensing of Forest Structure and GPS Telemetry Data Provide Insights on Winter Habitat Selection of European Roe Deer. *Forests*, 5, 1374-1390.

Johnson, C. J., K. L. Parker, D. C. Heard, and M. P. Gillingham. 2002. Movement parameters of ungulates and scale-specific responses to the environment. Journal of Animal Ecology 71:225-235.

Northrup, J. M., M. B. Hooten, C. R. Anderson, and G. Wittemyer. 2013. Practical guidance on characterizing availability in resource selection functions under a use–availability design. Ecology 94:1456-1463.

Sibly, R. M., H. M. R. Nott, and D. J. Fletcher. 1990. Splitting behavior into bouts. Animal Behaviour 39:63-69.

Wood, S.N. 2011. Fast stable restricted maximum likelihood and marginal likelihood estimation of semiparametric generalized linear models. Journal of the Royal Statistical Society (B) 73(1):3-36.

Wood, S.N. 2011. Generalized Additive Models: An Introduction with R. Second edition. Chapman and Hall/CRC Press. 2006.**Table S1** Model specifications for risk avoidance in roe deer. To model tradeoffs in risk avoidance between humans and lynx we built step selection functions using conditional logistic regression and compared model parameters between an all data model and a no-hunting interpolation. PR: predation risk by lynx (RSF in Table S2). HT: Habitat type. DOY: day of year. TOD: Time of day.

| **Roe deer SSF** |
| --- |
|  |
| **Habitat variables** |
| Predation risk |
| Habitat type (HT) |
| Edge distance |
| Altitude |
| Altitude^2^ |
| Slope |
| Slope^2^ |
| House density |
| Road distance |
| Southern exposition |
| **Temporal interactions** |
| PR:TOD |
| PR:DOY |
| HT:TOD |
| HT:DOY |
| PR:TOD |
| PR:DOY |
| Edge_dist:TOD |
| Slope:TOD |
| House_density:TOD |
| Road_dist:TOD |
| Altitude:DOY |
| Southern exp:DOY |

**Table S2** Model specifications for habitat selection (resource selection function) and activity patterns (logit model) in lynx. The habitat selection model is used as a proxy for predation risk in the roe deer step selection function (Table S1). The logit regression for lynx activity modelled the probability of a lynx being active given the model parameters. To test for an effect of hunting on lynx activity we compared an all data model with a no-hunting interpolation. A 1 denotes inclusion of a particular model parameter. HT: Habitat type. DOY: day of year. TOD: Time of day. The habitat selection model for the lynx is described in detail in Gehr (2016).

|  | **Lynx** | |
| --- | --- | --- |
|  | RSF | Logit |
| **Habitat variables** |  |  |
| Habitat type (HT) | 1 | 1 |
| Edge distance | 1 | 1 |
| Altitude | 1 | 1 |
| Altitude^2^ | 1 | 1 |
| Slope | 1 | 1 |
| Slope^2^ | 1 | 1 |
| House density | 1 | 1 |
| Road distance | 1 | 1 |
| Southern exposition | 1 | 1 |
| **Time variables** |  |  |
| DOY | - | 1 |
| TOD | - | 1 |
| **Spatial interactions** |  |  |
| HT:edge distance | 1 | 1 |
| House density:Road distance | 1 | 1 |
| **Temporal interactions** |  |  |
| HT:TOD | 1 | 1 |
| HT:DOY | - | - |
| Slope:TOD | 1 | 1 |
| House_density:TOD | 1 | 1 |
| Road_distabce:TOD | 1 | 1 |
| Altitude:DOY | 1 | 1 |
| Southern exposition:DOY | 1 | 1 |

**Table S3** Model output for the roe deer step selection function models as listed in Table S1 and described in detail in the main text. In the no-hunting interpolation we excluded the 10 week hunting period from the data and interpolated roe deer habitat use during the missing hunting period from the remaining data. To test for a hunting effect we compared the all data model to the non-hunting interpolation. Robust estimates of standard errors (se) are shown.

|  | All data model | |  | No hunting interpolation | |
| --- | --- | --- | --- | --- | --- |
| Variables | coef | se |  | coef | se |
| Habitat type | 0.03 | 0.01 |  | 0.09 | 0.02 |
| House density | -0.07 | 0.01 |  | -0.08 | 0.01 |
| Road distance | 0.21 | 0.01 |  | 0.21 | 0.02 |
| Edge distance | -0.26 | 0.01 |  | -0.26 | 0.01 |
| Southern exposition | 0.17 | 0.01 |  | 0.19 | 0.02 |
| Slope | 0.11 | 0.01 |  | 0.13 | 0.01 |
| Slope sq | -0.18 | 0.00 |  | -0.18 | 0.01 |
| Altitude | -0.42 | 0.02 |  | -0.40 | 0.03 |
| Altitude sq | -0.09 | 0.01 |  | -0.11 | 0.01 |
| Step length | 0.08 | 0.01 |  | 0.08 | 0.01 |
| Pred. risk | -0.18 | 0.02 |  | -0.25 | 0.03 |
| S.expo:ytsin | 0.22 | 0.02 |  | 0.19 | 0.03 |
| S.expo:ytcos | 0.25 | 0.02 |  | 0.25 | 0.02 |
| S.expo:ytsin2 | 0.11 | 0.02 |  | 0.12 | 0.02 |
| S.expo:ytcos2 | -0.10 | 0.02 |  | -0.14 | 0.03 |
| Slope:tsin | 0.10 | 0.01 |  | 0.10 | 0.01 |
| Slope:tcos | -0.13 | 0.01 |  | -0.13 | 0.01 |
| Altitude:ytsin | -0.19 | 0.03 |  | -0.24 | 0.05 |
| Altitude:ytcos | -0.05 | 0.03 |  | -0.03 | 0.03 |
| Altitude:ytsin2 | -0.17 | 0.03 |  | -0.18 | 0.03 |
| Altitude:ytcos2 | -0.01 | 0.03 |  | -0.03 | 0.04 |
| HT:ytsin | 0.13 | 0.02 |  | 0.01 | 0.03 |
| HT:ytcos | 0.04 | 0.02 |  | 0.05 | 0.02 |
| HT:ytsin2 | -0.04 | 0.02 |  | -0.09 | 0.02 |
| HT:ytcos2 | -0.18 | 0.02 |  | -0.28 | 0.03 |
| HT:tsin | -0.38 | 0.02 |  | -0.36 | 0.02 |
| HT:tcos | 0.58 | 0.02 |  | 0.56 | 0.02 |
| House:tsin | -0.23 | 0.01 |  | -0.22 | 0.01 |
| House:tcos | 0.17 | 0.01 |  | 0.17 | 0.01 |
| Road:tsin | 0.09 | 0.02 |  | 0.10 | 0.02 |
| Road:tcos | -0.03 | 0.02 |  | -0.05 | 0.02 |
| Edge:tsin | -0.22 | 0.01 |  | -0.22 | 0.01 |
| Edge:tcos | 0.37 | 0.01 |  | 0.37 | 0.01 |
| Pred.risk:ytsin | 0.03 | 0.02 |  | 0.14 | 0.03 |
| Pred.risk:ytcos | -0.12 | 0.02 |  | -0.16 | 0.02 |
| Pred.risk:ytsin2 | 0.11 | 0.02 |  | 0.13 | 0.02 |
| Pred.risk:ytcos2 | -0.07 | 0.01 |  | 0.00 | 0.02 |
| Pred.risk:tsin | -0.05 | 0.01 |  | -0.05 | 0.01 |
| Pred.risk:tcos | 0.03 | 0.01 |  | 0.05 | 0.01 |

**Table S4** Pearson correlations and relative importance values for each covariate of the all data step selection function and the no-hunting interpolation for roe deer. Lower correlation values indicate greater importance. We inferred relative importance by subtracting each correlation coefficient from 1 and then scaled the results by dividing each by the sum of all those differences. The average relative importance over 100 trials is reported here (see above).

|  | All data | |  | No hunting interpolation | |  |
| --- | --- | --- | --- | --- | --- | --- |
| Variables | r_pearson_ | rel. imp. |  | r_pearson_ | rel. imp. | |
| Habitat type | 0.9996 | 0.0002 |  | 0.9958 | 0.0019 |  |
| House density | 0.9893 | 0.0054 |  | 0.9871 | 0.0060 |  |
| Road distance | 0.8401 | 0.0798 |  | 0.8487 | 0.0705 |  |
| Edge distance | 0.8098 | 0.0950 |  | 0.8076 | 0.0897 |  |
| Southern exposition | 0.9819 | 0.0090 |  | 0.9781 | 0.0102 |  |
| Slope | 0.9774 | 0.0113 |  | 0.9709 | 0.0135 |  |
| Slope sq | 0.9233 | 0.0383 |  | 0.9229 | 0.0359 |  |
| Altitude | 0.6935 | 0.1530 |  | 0.7399 | 0.1212 |  |
| Altitude sq | 0.9781 | 0.0109 |  | 0.9757 | 0.0113 |  |
| Step length | 0.9720 | 0.0140 |  | 0.9727 | 0.0127 |  |
| Pred. risk | 0.8885 | 0.0557 |  | 0.7939 | 0.0961 |  |
| S.expo:ytsin | 0.9821 | 0.0089 |  | 1.0000 | 0.0000 |  |
| S.expo:ytcos | 0.9987 | 0.0007 |  | 0.9982 | 0.0008 |  |
| S.expo:ytsin2 | 0.9982 | 0.0009 |  | 0.9918 | 0.0038 |  |
| S.expo:ytcos2 | 0.9675 | 0.0162 |  | 0.9258 | 0.0346 |  |
| Slope:tsin | 0.8729 | 0.0634 |  | 0.8857 | 0.0533 |  |
| Slope:tcos | 0.7508 | 0.1244 |  | 0.7593 | 0.1122 |  |
| Altitude:ytsin | 0.9440 | 0.0279 |  | 0.9515 | 0.0226 |  |
| Altitude:ytcos | 0.9737 | 0.0132 |  | 0.9724 | 0.0129 |  |
| Altitude:ytsin2 | 0.9876 | 0.0062 |  | 0.9869 | 0.0061 |  |
| Altitude:ytcos2 | 0.9987 | 0.0007 |  | 0.9968 | 0.0015 |  |
| HT:ytsin | 0.9472 | 0.0264 |  | 0.9513 | 0.0227 |  |
| HT:ytcos | 0.7386 | 0.1301 |  | 0.7453 | 0.1183 |  |
| HT:ytsin2 | 0.9732 | 0.0134 |  | 0.9825 | 0.0082 |  |
| HT:ytcos2 | 0.9789 | 0.0105 |  | 0.9774 | 0.0105 |  |
| HT:tsin | 0.9944 | 0.0028 |  | 0.9931 | 0.0032 |  |
| HT:tcos | 0.9949 | 0.0026 |  | 0.9901 | 0.0046 |  |
| House:tsin | 0.9908 | 0.0046 |  | 0.9910 | 0.0042 |  |
| House:tcos | 0.9840 | 0.0080 |  | 0.9855 | 0.0068 |  |
| Road:tsin | 0.9568 | 0.0216 |  | 0.9488 | 0.0239 |  |
| Road:tcos | 0.9973 | 0.0013 |  | 0.9987 | 0.0006 |  |
| Edge:tsin | 0.9678 | 0.0161 |  | 0.9646 | 0.0165 |  |
| Edge:tcos | 0.9999 | 0.0000 |  | 0.9989 | 0.0005 |  |
| Pred.risk:ytsin | 0.9984 | 0.0008 |  | 0.9481 | 0.0242 |  |
| Pred.risk:ytcos | 0.9833 | 0.0083 |  | 0.9626 | 0.0174 |  |
| Pred.risk:ytsin2 | 0.9776 | 0.0112 |  | 0.9649 | 0.0164 |  |
| Pred.risk:ytcos2 | 0.9919 | 0.0040 |  | 1.0000 | 0.0000 |  |
| Pred.risk:tsin | 0.9958 | 0.0021 |  | 0.9954 | 0.0022 |  |
| Pred.risk:tcos | 0.9979 | 0.0011 |  | 0.9937 | 0.0029 |  |

**Table S5** Model output for the activity pattern models for lynx (probability of a lynx being active) as listed in Table S2 and described in detail in the main text. In the no-hunting interpolation we excluded the 10 week hunting period from the data and interpolated roe deer habitat use during the missing hunting period from the remaining data. To test for a hunting effect we compared the all data model to the non-hunting interpolation. Robust estimates of standard errors (se) are shown.

|  | Lynx models | | | | |
| --- | --- | --- | --- | --- | --- |
|  | All data | |  | No hunting interpolation | |
| Variables | coef | se |  | coef | se |
| Intercept | 0.066 | 0.066 |  | -0.110 | 0.088 |
| Habitat type | 0.600 | 0.077 |  | 0.728 | 0.102 |
| House density | 0.079 | 0.034 |  | 0.080 | 0.036 |
| Road density | -0.078 | 0.039 |  | -0.083 | 0.041 |
| Altitude | 0.092 | 0.049 |  | -0.004 | 0.064 |
| Altitude sq | 0.059 | 0.027 |  | 0.068 | 0.029 |
| Slope | -0.118 | 0.027 |  | -0.101 | 0.028 |
| Slope sq | -0.016 | 0.014 |  | -0.020 | 0.015 |
| Edge distance | 0.136 | 0.108 |  | 0.077 | 0.112 |
| Southern exposition | -0.126 | 0.072 |  | 0.047 | 0.103 |
| HT:Edge distance | -0.169 | 0.117 |  | -0.104 | 0.122 |
| House density:Road dist. | -0.021 | 0.033 |  | -0.002 | 0.035 |
| tsin | 0.034 | 0.034 |  | 0.051 | 0.036 |
| tcos | 0.302 | 0.060 |  | 0.308 | 0.063 |
| ytsin | -0.187 | 0.064 |  | 0.126 | 0.117 |
| ytcos | 0.083 | 0.080 |  | 0.013 | 0.090 |
| ytsin2 | -0.202 | 0.064 |  | -0.084 | 0.080 |
| ytcos2 | -0.020 | 0.062 |  | 0.165 | 0.082 |
| HT:tsin | -0.156 | 0.054 |  | -0.187 | 0.057 |
| HT:tcos | 0.338 | 0.086 |  | 0.295 | 0.089 |
| HT:ytsin | -0.096 | 0.080 |  | -0.282 | 0.137 |
| HT:ytcos | 0.117 | 0.101 |  | 0.176 | 0.112 |
| HT:ytsin2 | 0.139 | 0.080 |  | 0.057 | 0.096 |
| HT:ytcos2 | -0.086 | 0.083 |  | -0.175 | 0.107 |
| House:tsin | -0.121 | 0.029 |  | -0.129 | 0.031 |
| House:tcos | 0.104 | 0.042 |  | 0.119 | 0.043 |
| Road:tsin | -0.003 | 0.029 |  | -0.014 | 0.031 |
| Road:tcos | -0.018 | 0.042 |  | -0.021 | 0.044 |
| Altitude:ytsin | -0.065 | 0.054 |  | 0.104 | 0.081 |
| Altitude:ytcos | -0.035 | 0.060 |  | -0.076 | 0.066 |
| Altitude:ytsin2 | -0.098 | 0.050 |  | -0.024 | 0.060 |
| Altitude:ytcos2 | 0.031 | 0.049 |  | 0.124 | 0.059 |
| Slope:tsin | 0.108 | 0.027 |  | 0.120 | 0.029 |
| Slope:tcos | -0.043 | 0.036 |  | -0.032 | 0.037 |
| S.expo:ytsin | 0.125 | 0.094 |  | -0.175 | 0.154 |
| S.expo:ytcos | -0.120 | 0.100 |  | -0.015 | 0.114 |
| S.expo:ytsin2 | 0.083 | 0.088 |  | -0.062 | 0.104 |
| S.expo:ytcos2 | -0.001 | 0.087 |  | -0.157 | 0.111 |

**Table S6** Generalized cross validation (GCV) scores of the 4 GAM models for the systematic search data and the public reporting data, respectively. For the hunting model the full data set was used whereas for the no-hunting model the data during the hunting season was omitted and the model interpolated the predicted lynx kill rate for that time period based on the data before and after the hunting season. The model with the smallest GCV was selected which was in all 4 cases the model with dimension parameter k=16.

|  | **Systematic search data** | | **Public reporting data** | |
| --- | --- | --- | --- | --- |
| k | Hunting model | No hunting model | Hunting model | No hunting model |
| 10 | 0.0099 | 0.0116 | 0.0053 | 0.0034 |
| 12 | 0.0068 | 0.0023 | 0.0046 | 0.0018 |
| 14 | 0.0039 | 0.0049 | 0.0044 | 0.0020 |
| 16 | 0.0027 | 0.0019 | 0.0038 | 0.0015 |

**Figure S1** Map of the study area in the North-Western Alps of Switzerland including all GPS locations for lynx (orange) and roe deer (green) respectively.


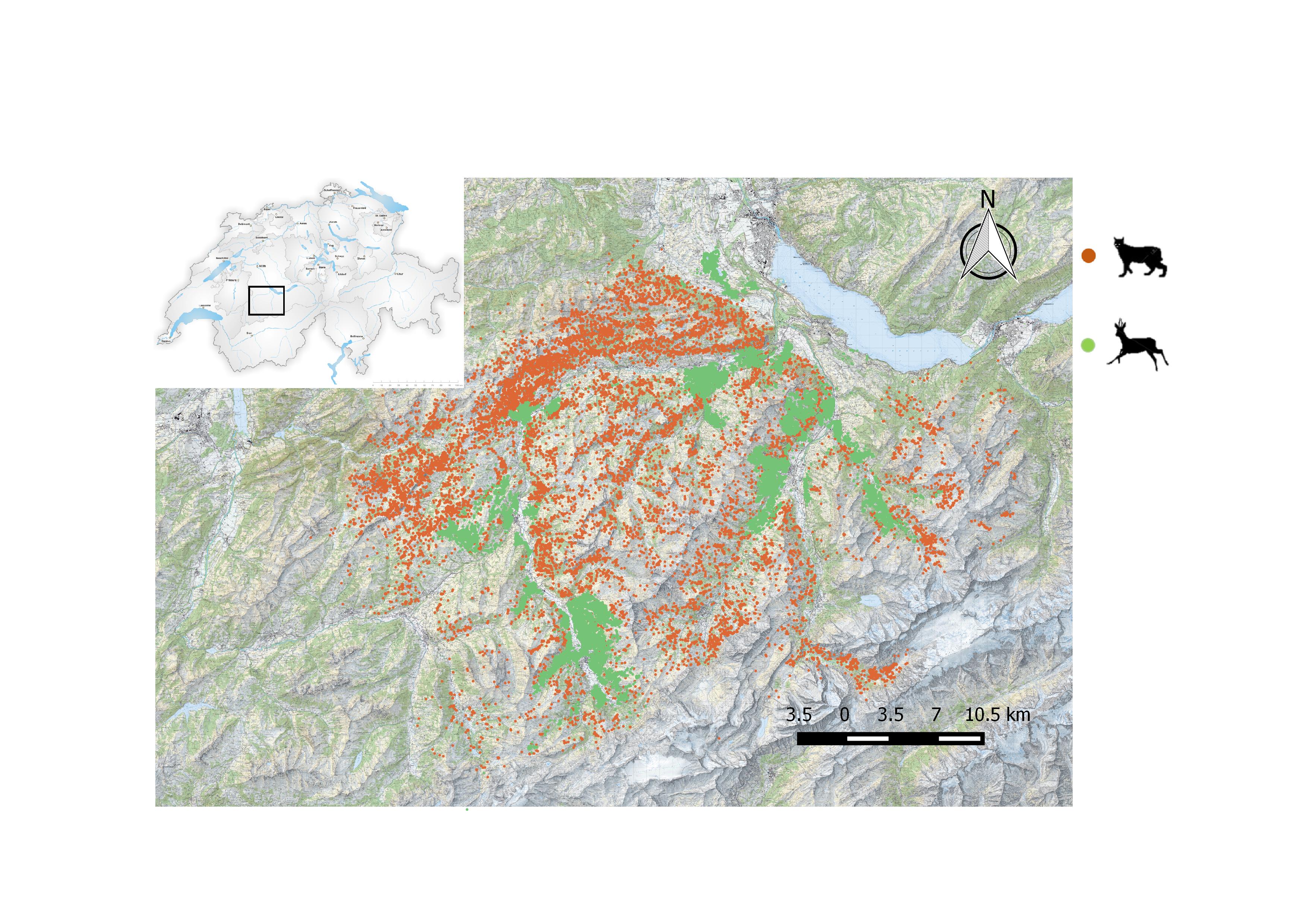


**Figure S2** Plot of the acceleration data used to separate deer steps into active and passive states (ACC; in millivolts) for individual 2249 on August 16 2012. Blue bars denote the acceleration raw data (in millivolt) whereas red shows the moving average for a window size of 2 (2x5 minutes). The black dotted line indicates the 10 millivolt threshold that was used for assigning the activity state. The horizontal black bars denote the active periods with a minimum length of 10 minutes. Blue and green dots show the GPS locations for passive and active periods respectively.


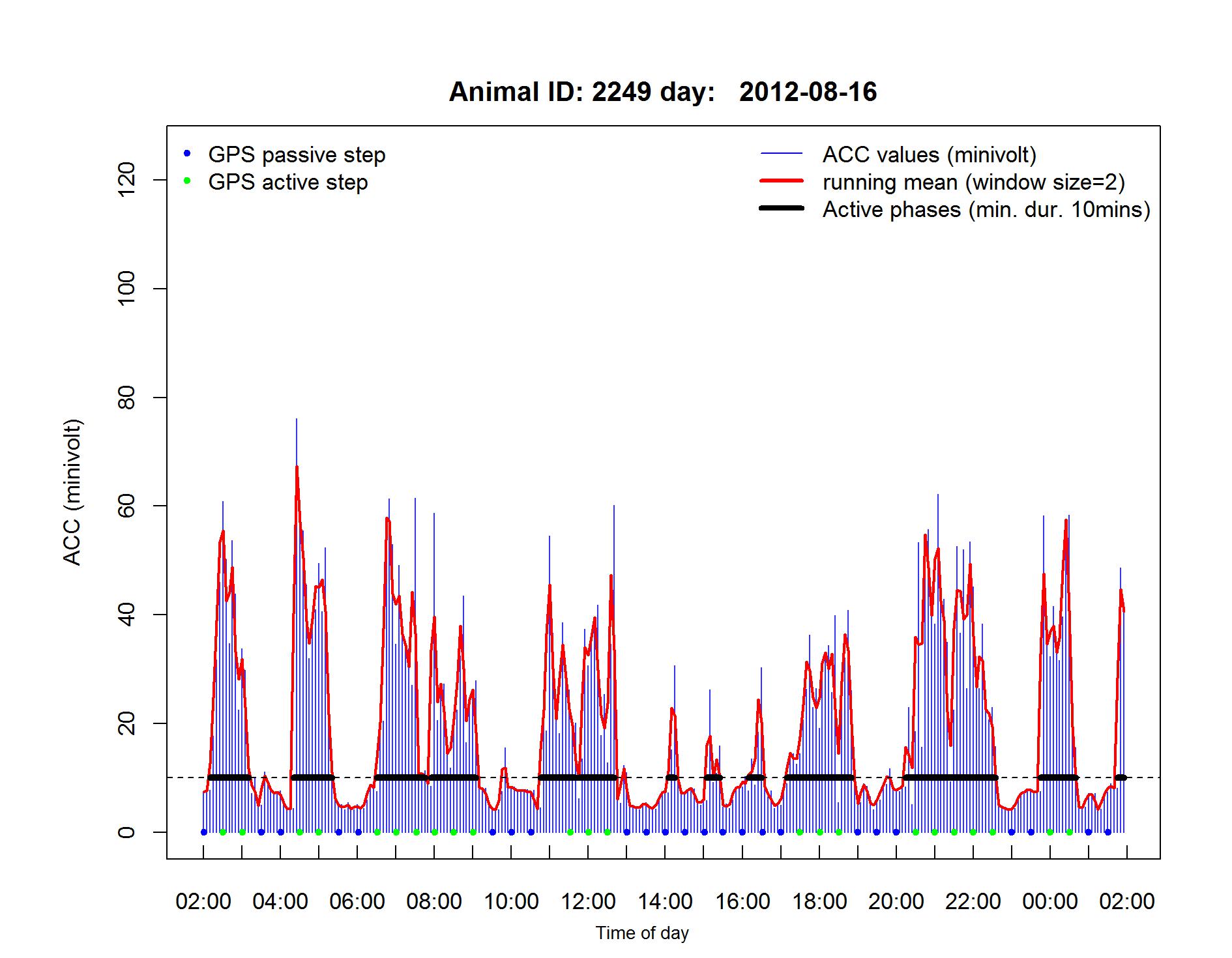


**Figure S3** Broken stick model (a) and dependency of movement rate on GPS fix interval (b). The log-frequencies of steps in each movement rate category are plotted against the movement rate categories (a). Results for the longer fix intervals (3-6h) are shown here. The two red lines indicate the two best linear model fits for the slow and fast movement process respectively. Dependency of movement rate on GPS fix interval (b). The red line indicates a linear model fit to the data.
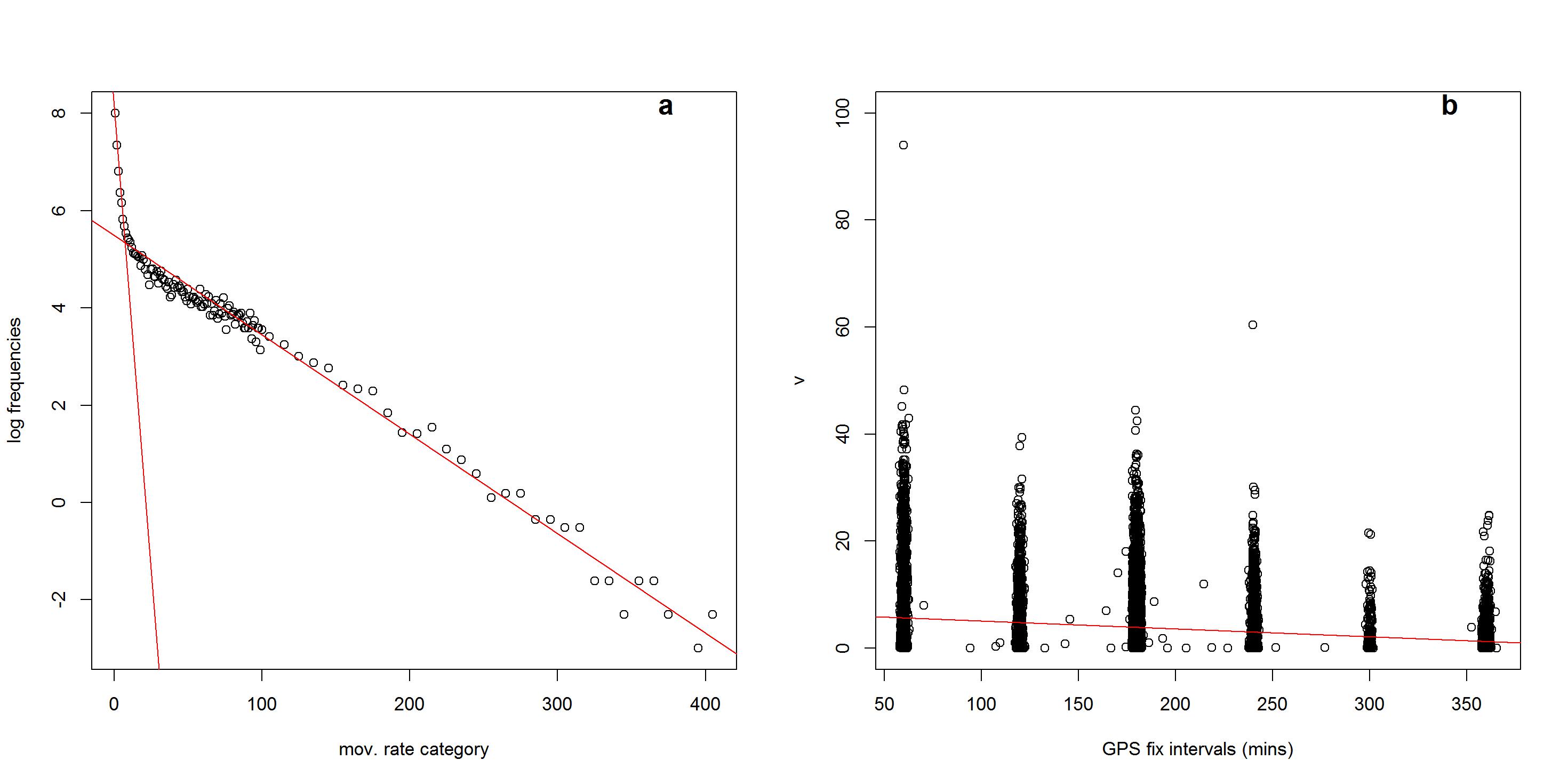


**Figure S4** Selection of altitude (a) and southern exposed slopes (b). Blue curves show the w(**x**)=exp(coef) values of the SSF using all data whereas green curves indicate w(**x**) for the non-hunting interpolation. The colored shaded areas denote the robust 95% -pointwise confidence intervals for the all data model (blue) and the non-hunting interpolation model (green). To calculate the effect displays, all covariates were set to their mean value except for altitude (a) and southern exposed slopes (b). The response to altitude denotes the avoidance/selection of high altitudes (75% quantile) relative to the mean altitude over the course of the year. The response to southern exposed slopes denotes the avoidance/selection of southern exposed slopes relative to other expositions over the course of the year. The shaded area in grey depict the 10 week hunting period in fall. The dotted line for w(**x**)=1 represents no avoidance/selection.


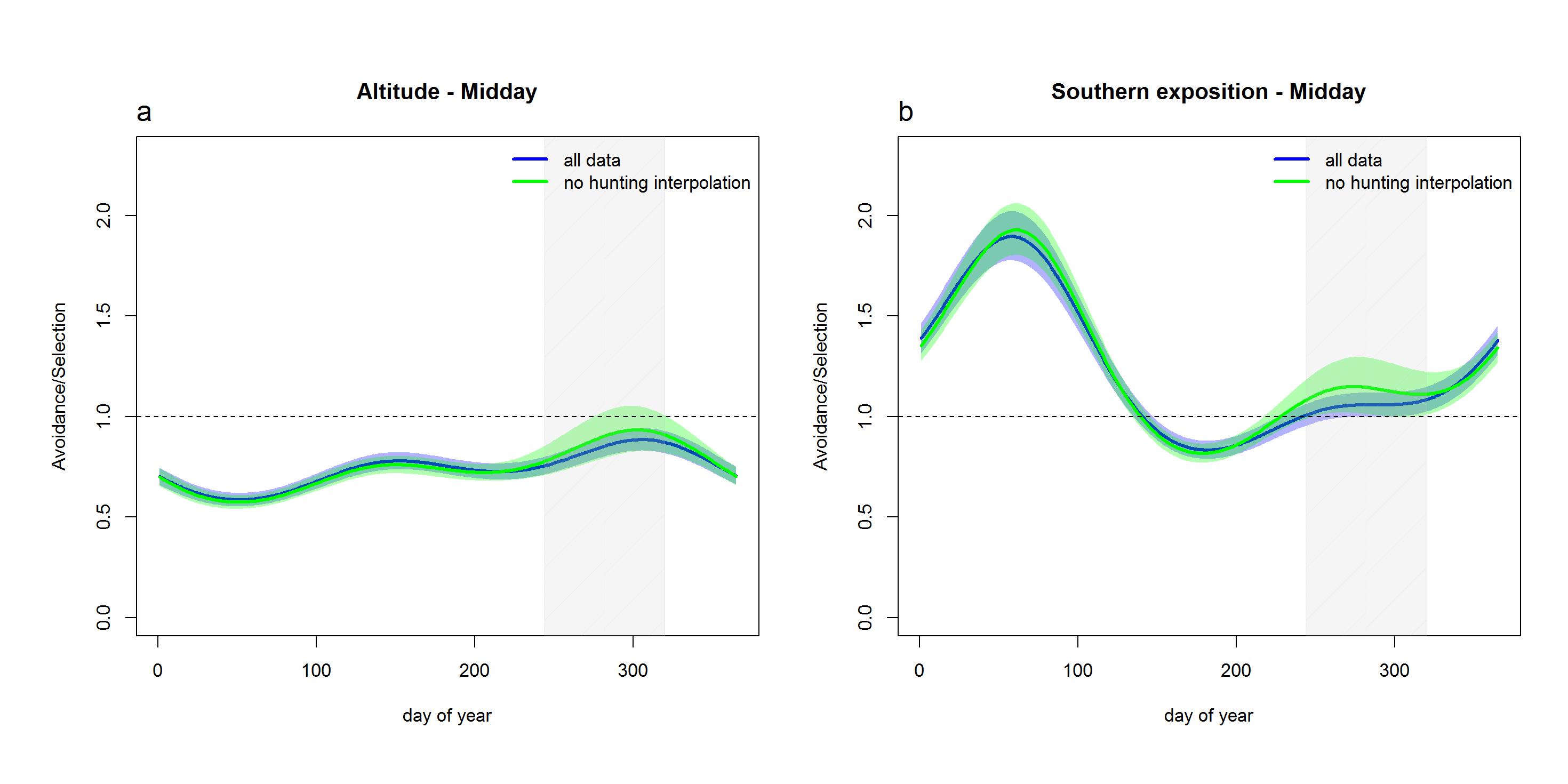


**Figure S5** Activity patterns of lynx inside the forest (a) and in open habitat (b) during nighttime over the course of the year. The results show the probability of a lynx being active while setting all other covariates to their mean. Blue curves show the activity for the all data model, green curves for the non-hunting interpolation model. The colored shaded areas denote the robust 95% -pointwise confidence intervals for the all data (blue) and the non-hunting interpolation model (green). The shaded area in grey depicts the 10 week hunting period in the fall.


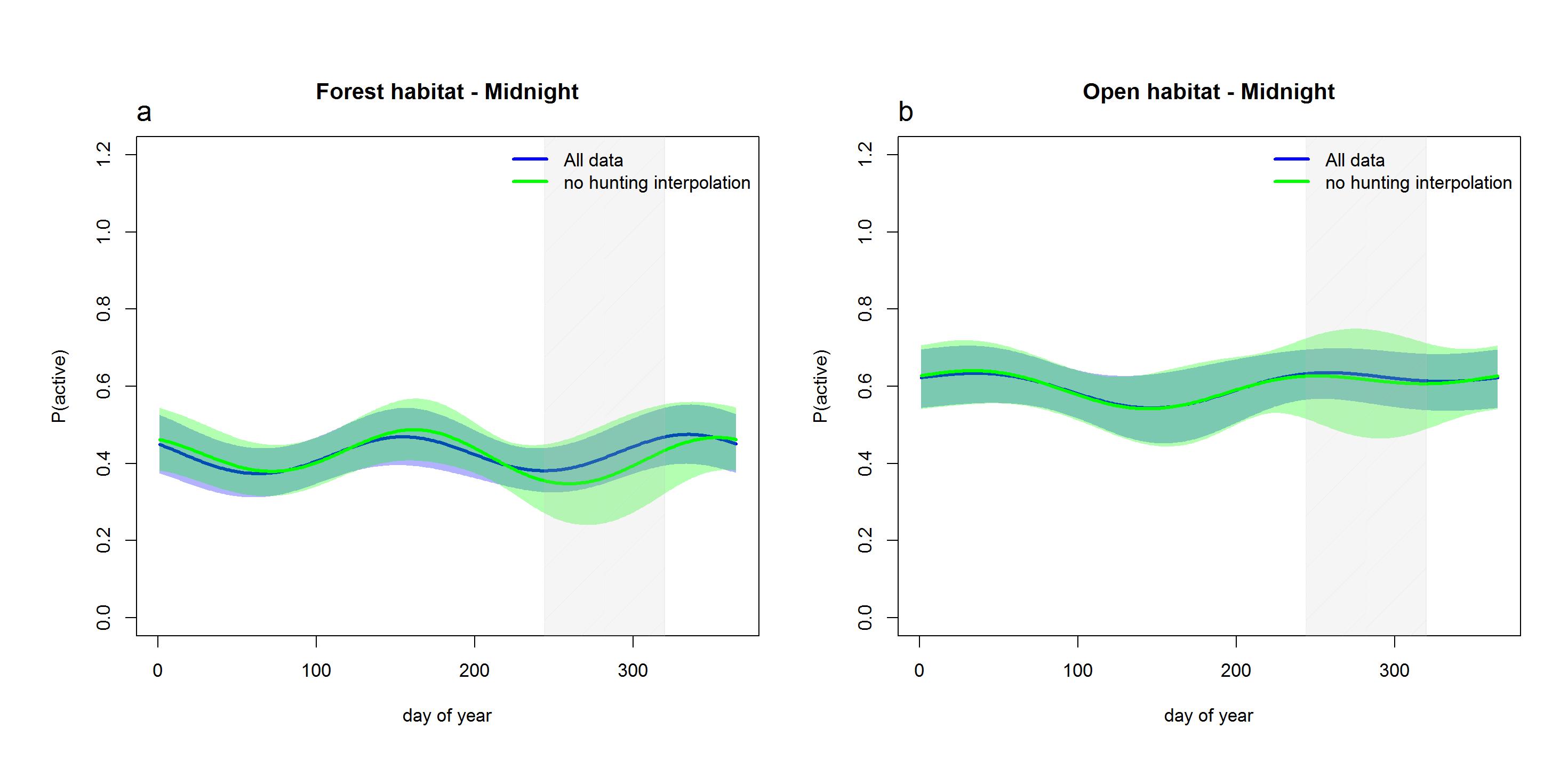

Supplement: Supplementary file 1 [file ECE3-8-109-s001.docx]
