# Supplementary material for: Hunting‐mediated predator facilitation and superadditive mortality in a European ungulate
Source: Ecol Evol. 2017 Nov 23;8(1):109–19. doi: 10.1002/ece3.3642 (PMC5756843; doi:10.1002/ece3.3642)
Supplement: Supplementary file 2 [file ECE3-8-109-s002.pdf]

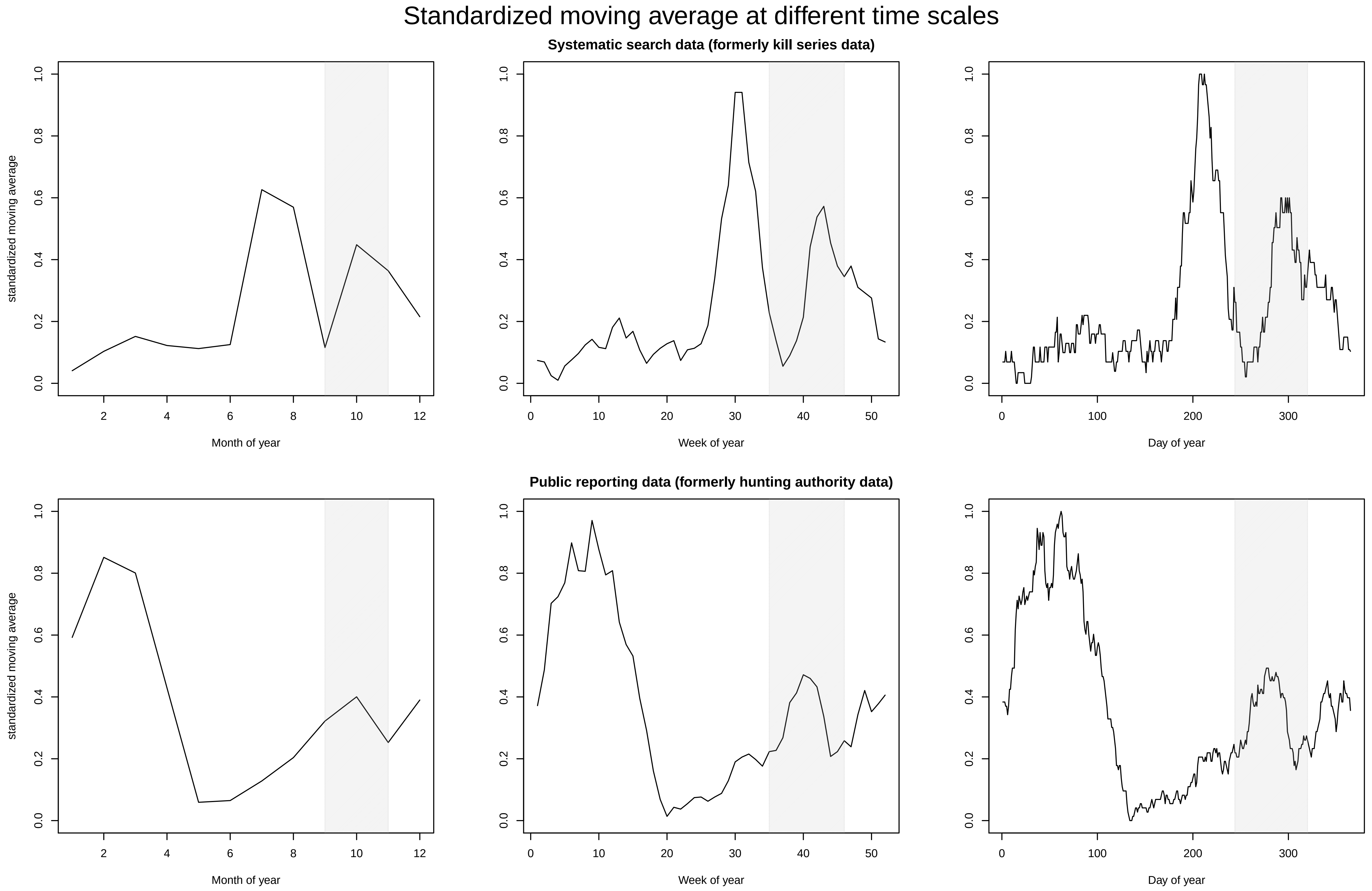

Standardized moving average at different time scales
Systematic search data (formerly kill series data)
Public reporting data (formerly hunting authority data)
standardized moving average
Month of year
Week of year
Day of year
0.0
0.2
0.4
0.6
0.8
1.0
2
4
6
8
10
12
0
10
20
30
40
50
100
200
300
